# A 37-year-old woman presenting with impaired visual function during antituberculosis drug therapy: a case report

**DOI:** 10.1186/1752-1947-5-317

**Published:** 2011-07-19

**Authors:** Abdulkabir A Ayanniyi, Rashidat O Ayanniyi

**Affiliations:** 1 Department of Ophthalmology, College of Health Sciences, University of Abuja, Abuja, Nigeria; 2 Department of Ophthalmology, University Teaching Hospital, Ado Ekiti, Nigeria; 3 Department of Pharmacology and Therapeutics, College of Health Sciences, University of Ilorin, Ilorin, Nigeria

## Abstract

**Introduction:**

Combination antituberculosis drug therapy remains the mainstay of treating tuberculosis. Unfortunately, antituberculosis drugs produce side effects including (toxic) impaired visual function, which may be irreversible. We report a case of antituberculosis-drug-induced impaired visual function that was reversed following early detection and attention.

**Case presentation:**

A 37-year-old Yoruba woman, weighing 48 kg, presented to our facility with impaired visual functions and mild sensory polyneuropathy in about the fourth month of antituberculosis treatment. Her therapy comprised ethambutol 825 mg, isoniazid 225 mg, rifampicin 450 mg, and pyrazinamide 1200 mg. Her visual acuity was 6/60 in her right eye and 1/60 in her left eye. She had sluggish pupils, red-green dyschromatopsia, hyperemic optic discs and central visual field defects. Her intraocular pressure was 14 mmHg. Her liver and kidney functions were essentially normal. Screening for human immunodeficiency virus was not reactive. Her impaired visual function improved following prompt diagnosis and attention, including the discontinuation of medication.

**Conclusions:**

The ethambutol and isoniazid in antituberculosis medication are notorious for causing impaired visual function. The diagnosis of ocular toxicity from antituberculosis drugs should never be delayed, and should be possible with the patient's history and simple but basic eye examinations and tests. Tight weight-based antituberculosis therapy, routine peri-therapy visual function monitoring towards early detection of impaired function, and prompt attention will reduce avoidable ocular morbidity.

## Introduction

Tuberculosis is a multisystemic and wide-ranging communicable disease across the globe, specifically in resource limited economies. It is caused by a type of acid-fast and alcohol-fast mycobacteria. The bacilli are difficult to treat and there may be resistance, which is common in immunosuppressed states, especially human immunodeficiency virus (HIV) and/or acquired immunodeficiency syndrome (AIDS). However, the 'combination drug therapy' employed in the treatment of tuberculosis has been hailed as a medical breakthrough.

Various combination drug regimens are employed in the treatment of tuberculosis. The common combination drug therapy is one comprising the 'first-line' antituberculosis drugs including ethambutol, isoniazid, rifampicin and pyrazinamide [1].

Unfortunately, these drugs, despite being effective in treating tuberculosis, produce a number of side effects, including impaired visual function [1]. For example, ethambutol is notorious for causing optic neuropathy and chiasmopathy [2,3]. Furthermore, and disturbingly, ethambutol is toxic to retinal ganglion cells via an excitotoxic pathway [4]. Additionally, Isoniazid has been implicated in optic neuropathy as well as peripheral neuropathy and hepatotoxicity [1,5]. Rifampicin is notorious for micosomal enzyme induction and hepatotoxicity, while pyrazinamide has been associated with hepatotoxicity, gastrointestinal upset and hyperuricemia [1].

The adverse effects of combination antituberculosis medication may be related to drug dose and duration of use [1] or may be idiosyncrasies.

Additionally, impaired renal or liver function can lead to adverse drug effects by way of impaired drug metabolism.

This report highlights the sight restoration, following early detection and attention, in a 37-year-old woman who developed impaired visual function after four months on high-dosage combination antituberculosis therapy. Recommendations on routine vision screening and monitoring of patients on antituberculosis drugs towards early detection and attention are discussed.

## Case report

**Figure 1:**
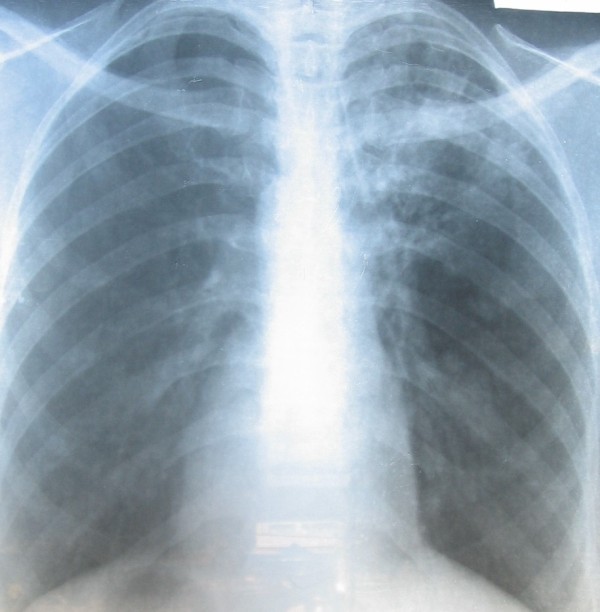
**Chest radiograph of our patient on presentation to our clinic**.

Our patient was a 37-year-old, 48 kg Yoruba woman, who developed impaired visual function while on treatment for pulmonary tuberculosis. She was diagnosed as having pulmonary tuberculosis based on a history of chronic cough, weight loss, positive acid- and alcohol-fast bacilli sputum examinations and reticulonodular chest features of pulmonary tuberculosis evident on radiology (Figure 1).

She successfully completed a two-month intensive treatment course on a daily four-drug combination comprising ethambutol 825 mg, isoniazid 225 mg, rifampicin 450 mg, and pyrazinamide 1200 mg. Our patient's weight improved to 52 kg. This two-month course was followed by a continuous phase of treatment. This comprised a daily two-drug combination including ethambutol 825 mg and isoniazid 450 mg. The two-drug combination was, however, substituted with only rifampicin about 11 weeks into the continuous phase when our patient reported blurred vision at the chest clinic where she was being managed. There was total withdrawal of the antituberculosis drug about two weeks later on account of persistent impaired visual function. By then, our patient had been receiving antituberculosis therapy for a period of five months, including the two-month intensive and three-month continuous phases.

She presented to our eye clinic about nine days after her antituberculosis drugs had been discontinued on account of her progressive painless diminishing vision of approximately one month's duration. Our patient also complained of a tingling sensation in her lower limbs. There was no record of a visual assessment before and during her therapy, however, our patient stated she had had normal vision previously. She had no family history of significant blinding ocular conditions and did not wear glasses. Furthermore, our patient had no history suggestive of diabetes mellitus, hypertension, sickle cell disease or HIV and/or AIDS.

**Figure 2:**
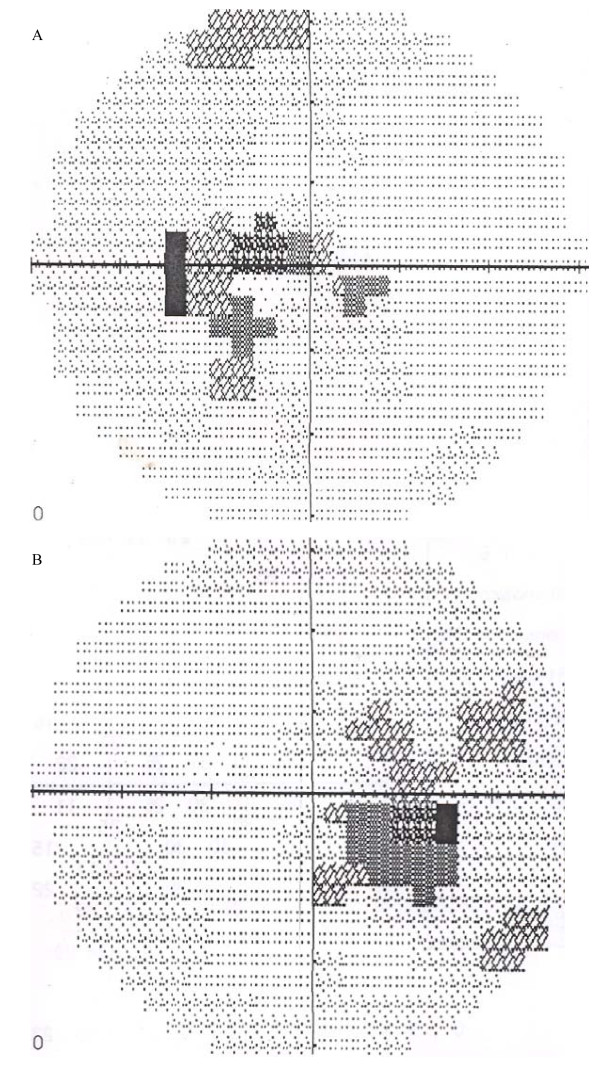
**CVF (a) left eye and (b) right eye, during impaired visual function**.

A physical examination on presentation showed a wasted and concerned patient, however her vital signs were normal. Her visual acuity (VA) was 6/60 in her right eye and 1/60 in her left eye. She had red-green dyschromatopsia using Ishihara color plates and her pupils were sluggish in their response to light. Her optic discs were hyperemic. Her intraocular pressure was 14 mmHg in both eyes. She had central visual field (CVF) defects (Figure 2a, b). Biochemical tests to assess kidney and liver function were essentially normal except for elevated alkaline phosphatase (Table 1). A screening for HIV was not reactive.

**Table 1:** Blood biochemical parameters in our patient

Biochemical parameter	Normal reference value	Patient value
Bilirubin total, μmol/L	Up to 20	1.8

Bilirubin conjugated, μmol/L	5	0.5

Protein total, g/L	58 to 80	70.0

Albumin, g/L	35 to 50	42.0

Creatinine, μmol/L	50 to 110	90.0

Bicarbonate, μmol/L	20 to 30	26.0

Chloride, mmol/L	95 to 110	104.0

Potassium, mmol/L	3 to 5	3.8

Sodium, mmol/L	120 to 140	129.0

Urea, mmol/L	1.7 to 9.1	1.9

Alkaline phosphatase, IU	21 to 107	138.0

Alanine transferase, IU	4 to 18	7.0

Aspartate transferase, IU	Up to 22	4.0

**Figure 3:**
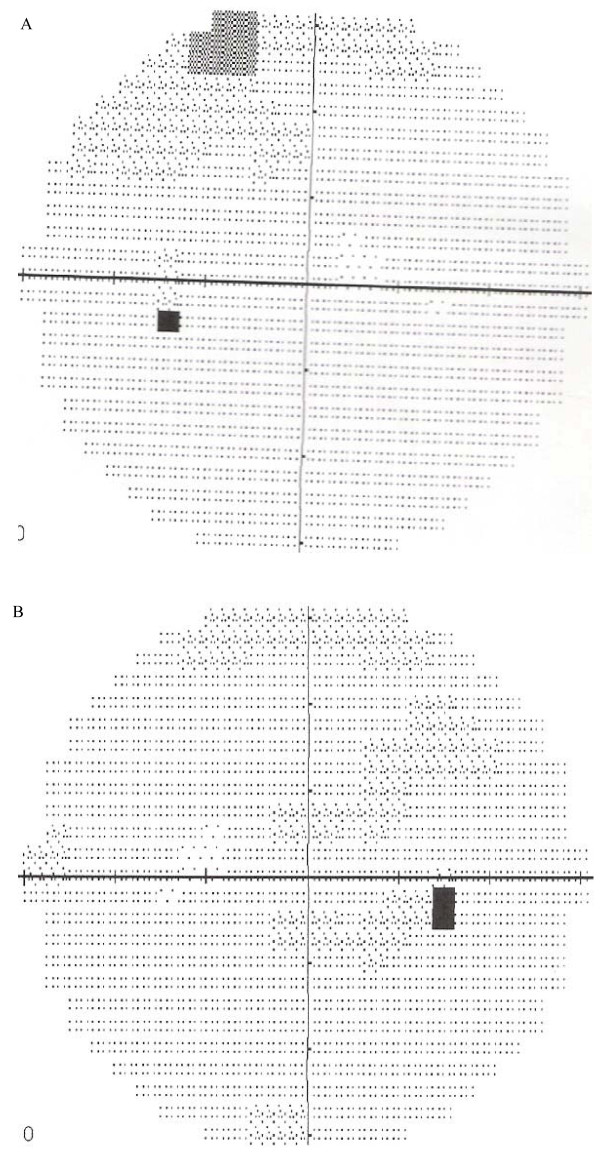
**CVF (a) left eye and (b) right eye, months after discontinuation of drugs**.

Except for multivitamins (Dolo-Neurobion^® ^taken orally daily) taken by our patient for three weeks, our patient was not on any other medication. She was counseled and reassured of a high chance of visual recovery over time. Our patient was monitored in our eye clinic at two-weekly intervals. At each visit, aside from our patient's briefings on visual function, her VA, color vision, pupillary activities and funduscopy were checked and documented. Her VA initially got worse, declining to 1/60 in both eyes two weeks after initial presentation, but later improved steadily following the discontinuation of the antituberculosis therapy. Our patient was last reviewed by our eye clinic nine months after her initial presentation, and her VA was recorded as unaided VA right eye 6/24-1, left eye 6/12+2 and aided (using correction with lenses; right eye -2.75DS, left eye -1.00DS) VA right eye 6/9-2, left eye 6/6-3. A repeat CVF test (Figure 3a, b) performed eight months after her initial CVF showed that the CVF defects had disappeared.

## Discussion

Antituberculosis drugs, as with many other drugs, produce unwanted side effects, especially when used at high dosages and usually for periods of more than two months [2,5]. The toxic effects can involve many organs, including the eyes, kidneys, liver, and blood tissues.

Our patient had normal hepatic and renal function on antituberculosis medication, as revealed by her normal biochemical indices. This suggests that the drug toxicity was not related to impaired liver or renal function. The screening for HIV in our patient, though negative, was desirable, as tuberculosis can coexist with HIV/AIDS [6]. Our patient was in the sexually active catchment age where HIV/AIDS is common.

Of the four drugs in the combination antituberculosis therapy, both ethambutol and isoniazid are capable of causing the impaired visual function seen in our patient. Ethambutol, especially, is notorious for causing optic neuropathy, chiasmopathy, and retinal toxicity. The dose of ethambutol as used in our patient was above 15 mg/kg, a level at which ocular toxicity is common [5]. However, vision loss even with much lower doses is not impossible in idiosyncratic situations. Furthermore, our patient had used both ethambutol and isoniazid for more than four months, a duration sufficient to produce ocular toxicity [5].

The neurovitamins (Dolo-Neurobion including vitamin B6) were expected to augment visual recovery. However, restoration of impaired visual function may have been achieved without neurovitamin supplements once the suspected toxic agent was discontinued, and of course before damage became irreversible. Our patient had a sudden onset of diminishing visual acuity that gradually improved over a period of months after discontinuation of medication and was not necessarily based on any specific medication, including the use of neurovitamins. Furthermore, the background fundal findings of hyperemic optic discs, red-green dyschromatopsia, and sluggish pupillary activity in our patient, as well as CVF defects, all reinforced our suspicion of toxic neuropathy, specifically caused by ethambutol and worsened by isoniazid.

The relevant history of impaired visual functioning while on antituberculosis drugs including ethambutol and isoniazid, and basic eye examinations and tests were sufficient for accurate diagnosis and hence suitable intervention to save the sight in our patient. This approach should be sufficient in saving the sight of patients on antituberculosis medication elsewhere as well. This benefit is further underscored as it can be achieved without sophisticated medical tests, which are not readily available in many societies. However, tests including multifocal electroretinogram (mfERG) and visual evoked potential (VEP) could suggest associated retinal toxicity in patients taking ethambutol. Results from mfERGs have been found to be abnormal in ethambutol-induced macular toxicity, and should therefore be useful in diagnosis and serial assessment of ethambutol-related ocular toxicity [7]. Furthermore, where available and affordable, tests such as magnetic resonance imaging (MRI) and optical coherence tomography (OCT) [8] could be employed to investigate ethambutol ocular toxicity. MRI scans of the optic nerves and chiasm, with normal findings in toxic and/or nutritional optic neuropathy, could be useful to differentiate between bilateral cecocentral scotomas and compressive or infiltrative lesions of the optic chiasm. OCT, being an objective test, could be used to quantify the loss of retinal nerve fibers from the optic nerves as a sign of early toxicity from ethambutol [8].

It was found that our patient, as in many others in resource-limited communities, had received no peri-therapy visual assessment. However, early report and detection, prompt referral, and prompt attention contributed to restoration of visual function in our patient. Apart from this, our patient benefited from a discontinuation of drugs, which of course remains the mainstay of treating ethambutol-associated and isoniazid-associated ocular toxicity [5].

Irreversible visual impairment as a consequence of antituberculosis medication may not be common, but it does complicate the management of tuberculosis and compromises patient quality of life. Increasing patient, health worker and facility preparedness for identifying and managing symptoms or signs of possible visual complications of ethambutol and isoniazid use may assist in preventing cases of irreversible visual impairment from antituberculosis medication.

Patients on antituberculosis medication (and their parents or guardians) may also benefit from health education on possible visual complications of ethambutol and isoniazid. Where possible, patients could be taught how to assess VA and how to perform color vision tests for self-monitoring of visual function. These charts could be made available for patients to use at home. Patients could then report to their clinic (in person or by telephone) in case of visual complaints or detected visual defects.

Furthermore, the attending health staff could be trained on the possible ocular side effects of ethambutol and isoniazid, including visual impairment and color vision defects. They should specifically ask patients about these at each clinic visit (screening). They should also carry out tests on patients, interpret VA and color vision test results for possible changes and advise discontinuation of drugs, promptly referring patients to eye care specialists.

Finally, involved health facilities should routinely assess patients for visual function while on antituberculosis drug therapy. This could be achieved by referring patients for visual function assessment before, during and after drug therapy. Alternatively, trained health staff in such facilities could be involved in peri-therapy visual assessment, as already mentioned. The required minimum for peri-therapy visual function assessment for patients on ethambutol and isoniazid drugs should be VA and color vision tests. The health facility could make visual acuity and color vision charts available.

Following the advocacy visit to the chest clinic, where our patient was being managed, the coordinator in charge of the tuberculosis program agreed to implement our above recommendations. The prompt referral of patients with ocular complaints and/or detected ocular abnormalities to eye care specialists can prevent ocular morbidity and/or mortality among patients on antituberculosis drugs.

## Conclusions

Ethambutol and isoniazid used in antituberculosis treatment are notorious for causing impaired visual function. A diagnosis of ocular toxicity from antituberculosis drugs should never be delayed, and should be possible with patient history and simple but basic eye examinations and tests. Tight weight-based antituberculosis therapy, routine peri-therapy visual function monitoring towards early detection of impaired functions, and prompt attention will reduce avoidable ocular morbidity among patients on antituberculosis drugs.

## Consent

Written informed consent was obtained from the patient for publication of this case report and any accompanying images. A copy of the written consent is available for review by the Editor-in-Chief of this journal.

## Competing interests

The authors declare that they have no competing interests.

## Authors' contributions

AAA came up with the study concept and design, and was responsible for patient care, drafting the manuscript and the literature review. ROA edited the manuscript, performed the literature review, and revised the manuscript. All authors read and approved the final manuscript.
